# Steering Evolution with Sequential Therapy to Prevent the Emergence of Bacterial Antibiotic Resistance

**DOI:** 10.1371/journal.pcbi.1004493

**Published:** 2015-09-11

**Authors:** Daniel Nichol, Peter Jeavons, Alexander G. Fletcher, Robert A. Bonomo, Philip K. Maini, Jerome L. Paul, Robert A. Gatenby, Alexander R.A. Anderson, Jacob G. Scott

**Affiliations:** 1 Department of Computer Science, University of Oxford, Oxford, United Kingdom; 2 Department of Integrated Mathematical Oncology, H. Lee Moffitt Cancer Center and Research Institute, Tampa, Florida, United States of America; 3 Wolfson Centre for Mathematical Biology, Mathematical Institute, University of Oxford, Oxford, United Kingdom; 4 Department of Medicine, Louis Stokes Department of Veterans Affairs Hospital, Cleveland Ohio, United States of America,; 5 School of Electrical Engineering and Computing Systems, University of Cincinnati, Cincinnati, Ohio, United States of America; Emory University, UNITED STATES

## Abstract

The increasing rate of antibiotic resistance and slowing discovery of novel antibiotic treatments presents a growing threat to public health. Here, we consider a simple model of evolution in asexually reproducing populations which considers adaptation as a biased random walk on a fitness landscape. This model associates the global properties of the fitness landscape with the algebraic properties of a Markov chain transition matrix and allows us to derive general results on the non-commutativity and irreversibility of natural selection as well as antibiotic cycling strategies. Using this formalism, we analyze 15 empirical fitness landscapes of *E. coli* under selection by different *β*-lactam antibiotics and demonstrate that the emergence of resistance to a given antibiotic can be either hindered or promoted by different sequences of drug application. Specifically, we demonstrate that the majority, approximately 70%, of sequential drug treatments with 2–4 drugs promote resistance to the final antibiotic. Further, we derive optimal drug application sequences with which we can probabilistically ‘steer’ the population through genotype space to avoid the emergence of resistance. This suggests a new strategy in the war against antibiotic–resistant organisms: drug sequencing to shepherd evolution through genotype space to states from which resistance cannot emerge and by which to maximize the chance of successful therapy.

## Author Summary

Increasing antibiotic resistance, coupled with the slowing rate of discovery of novel antibiotic agents, is a public health threat which could soon reach crisis point. Indeed, the last decade has seen the emergence of deadly, highly resistant forms of pathogens, such as *Escherichia coli*, *Acenitobacter baumanii*, *Klebsiella pneumoniae*, *Enterococcus* and *Staphylococcus aureus* as well as non–bacterial pathogens including malaria and viruses such as HIV. Here, we develop a mathematical model of an evolving bacterial population, which allows us to predict the probability of resistant strains emerging. Using this model we show how sequences of drugs can be prescribed in order to prevent resistance where each drug alone may fail. These model predictions suggest a novel treatment strategy: using sequences of antibiotics to ‘steer’ the evolution of a pathogen to a configuration from which resistance to a final antibiotic cannot emerge. Further, we test the likelihood of resistance emerging when arbitrary sequences of antibiotics are prescribed, finding that approximately 70% of arbitrary sequences of 2–4 drugs promote resistance to the final drug. This result serves as a cautionary warning that we may be inadvertently promoting resistance through careless (or random) prescription of drugs.

## Introduction

Resistance to antibiotic treatments within bacterial pathogens poses an increasing threat to public health, which, coupled with the slowing discovery of novel antibiotics, could soon reach crisis point [1, 2]. Novel classes of antibiotics discovered since 1987 are few in number [3]. Thus, it is becoming ever clearer that if we are to combat highly resistant bacterial infections, then we must find new ways to prevent resistance and new applications of existing antibiotics to these pathogens. Public health efforts have attempted to stem the emergence of resistance by reducing unnecessary prescription of antibiotics [4–6] and stopping the addition of sub-therapeutic antibiotics to livestock feed [7]. However, such policies require global adoption to be truly effective [8], which they have not yet achieved, and is likely infeasible.

Recently, there have been efforts to explore how existing antibiotics can be used in new ways to provide effective treatments for resistant pathogens, for example through combination therapy [9–11] or drug sequencing. The emergence of drug resistance is governed by Darwinian principles, with treatment imposing selective pressure on a population that selects for resistant mutants. It has been suggested (in the realm of cancer therapy, but with arguments equally applicable to treating bacterial infections) that we must design treatments which use sequences of drugs to account for, or even exploit, evolution [12]. The number of possible sequential treatments from even modest numbers of drugs is much too large for optimal therapies to be identified through an exhaustive experimental search. As such, the development of sequential treatment strategies has become a problem best approached with the aid of mathematical biology. Indeed, sequential therapies have previously been explored in the field of cancer research where early mathematical modeling tested drug sequences to derive the so–called “worst drug rule” for sequential cancer therapies [13, 14] (see [15] for a recent extension of their methods). In the thirty years since these results were published a number of mathematical models have been developed which attempt to design optimal sequential or combination therapies. Many of these techniques are applicable not only to cancers but also infectious disease (see [16–20] for reviews).

Goulart et al [21] used a search algorithm on graphs determined by fitness landscapes of *Escherichia coli* to design antibiotic cycling strategies—long term hospital–scale treatment protocols that cycle antibiotics in sequence over timescales of weeks, months or years to prevent resistance. However, our understanding of the mechanisms underlying these strategies remains limited and mathematical models which consider the entire space of possible strategies suggest that cycling may offer no significant benefit over mixed–drug strategies [22, 23]. These models have led the authors (and others [24, 25]) to suggest that more sophisticated techniques are needed to determine optimal therapies.

In order to understand how to minimize the emergence of resistant pathogens, and to decide how best to design sequential treatments, we must first understand how their evolution is driven by the selective pressures of different antibiotic drugs—a fundamental problem of biology [26]. In particular, if we understand which traits are likely to be selected for by which treatments, then we may be able to avoid selecting for those traits that confer resistance. Recent insights into the evolutionary process have yielded some actionable information. Weinreich et al [27, 28] showed that if the genome of a pathogen exhibits sign epistasis, where a given mutation is beneficial on some genetic backgrounds and deleterious on others, then there can exist inaccessible evolutionary trajectories. Further, Tan et al [29] studied the evolutionary trajectories of *E. coli* under different antibiotics and found that adaptive mutations gained under one antibiotic are often irreversible when a second is applied. Such irreversible paths can occur when resistance conferring mutations for one environment carry a cost in another that can be mitigated by other compensatory mutations [30, 31]. These findings lead us to hypothesize that one antibiotic could be used to irreversibly steer the evolution of a population of pathogens to a genotype (or combination of genotypes) which is sensitive to a second antibiotic but also from which it is unlikely to acquire resistance to that antibiotic. This hypothesis was partly verified by the work of Imamovic et al [32], who demonstrated that evolving *E. coli* to become resistant to certain antibiotics can increase sensitivity to others. However, those experiments did not exhaustively consider all evolutionary trajectories but instead highlighted only those that arose in a small number of replicates; further, the authors do not consider how evolution may proceed once a second drug is applied and whether resistance can then emerge.

### Model Overview

In this study we present a model that abstracts the evolutionary dynamics of an asexually reproducing population with unspecified, but variable, population size in which individuals are subject to point mutation at reproduction. We model the genotype of each individual as a length *N* sequence of 0s or 1s indicating the absence or presence of *N* known fitness conferring mutations of interest. In particular, our results are derived from a model of *E. coli* with genotypes of length *N* = 4, indicating the presence of four possible amino acid substitutions (specifically M69L, E104K, G238S and N276D) giving 2^4^ = 16 possible genotypes in total. The fitness values associated with individuals in this model are, in general, abstracted away from biological reality but in the context of this work are empirically determined by average growth rates of specific strains under different drugs (see [33] for details).

Our model builds on the well–studied Strong Selection Weak Mutation (SSWM) model derived by Gillespie [34, 35] which assumes that the disease population is isogenic and that evolution proceeds as the population genotype is periodically replaced by a fitter mutant. This model is valid under a broad range of circumstances provided that the mutation rate is not too high or the population size too small (a precise description of the necessary relationship between population size and mutation rate is provided in the Materials and Methods). The benefit of this abstraction is that, provided they fall within acceptable limits, we are able to ignore the population size and mutation rate in predicting evolutionary trajectories. This allows us to efficiently determine evolutionary trajectories and to consider trajectories either at the patient scale or at the whole clinic scale as in Goulart et al [21], although without explicit knowledge of the population parameters we are unable to predict the time taken to traverse these evolutionary trajectories.

Under the assumption of SSWM the evolutionary trajectory of a population can be viewed as a weighted random walk through genotype space which is determined by the fitness landscape imposed by a drug. Our work differs from previous models that utilize the SSWM model in that we encode this random walk formally as a Markov chain. This enables us to determine the probability of evolutionary trajectories to the fitness optima of landscapes through matrix multiplication. Specifically, we encode uncertainty about the current population genotype as a probability distribution vector *μ* with length equal to the number of genotypes. We can then determine the probability of reaching different fitness optima of a landscape by multiplying *μ* by a limit matrix that is determined by successive multiplication of the Markov chain transition matrix associated with that landscape. This encoding assumes that drugs are prescribed for sufficiently long for evolution to proceed to equilibrium in the fitness landscape.

Here, we present the construction of the Markov chain from fitness landscapes and use this construction to derive mathematical results regarding drug ordering and cycling from the algebraic properties of its transition matrix. In particular, we demonstrate that the order in which drugs are prescribed can have significant effects on the final population configuration—a phenomenon we call *non–commutativity* of selective pressures. Using previously measured landscapes for 15 *β*-lactam antibiotics we illustrate how the emergence of high resistance, which we take throughout the following to mean evolution to the highest fitness peak of the landscape, can be both hindered and promoted by different orderings of selective pressures. Finally, we exhaustively explore all possible ordered sequences of two, three and four antibiotics, finding that the majority, approximately 70%, of arbitrary drug sequences promote the emergence of resistance. These findings suggest new treatment strategies which use rational orderings of drugs to shepherd evolution through genotype space to a configuration that is sensitive to treatment, as in the work of Imamovic et al [32], but also from which resistance cannot emerge.

## Results

### Non-commutativity and Cycling of Natural Selection

Using the Markov chain model presented in the Materials and Methods, we can formally prove that for a large class of fitness landscape pairs there is non-commutativity in the evolutionary process as described by the SSWM assumptions. Suppose that there are two drugs, *X* and *Y*, with corresponding fitness landscapes *x* and *y*, and that we wish to determine what, if any, difference there is between applying *X* followed by *Y* to a population as opposed to applying *Y* followed by *X*. We can construct the Markov chain transition matrices *P*_*x*_ and *P*_*y*_ corresponding, respectively, to *x* and *y* according to Eq 3 and take the limits $P_{x}^{*}$ and $P_{y}^{*}$ of these matrices under successive multiplication. For a given initial population distribution vector *μ*, we can find the distribution over genotypes after evolution proceeds to equilibrium in the fitness landscapes by matrix multiplication. For example, the distribution after drug *X* is prescribed is given by $\mu^{\prime }=\mu P_{x}^{*}$. Hence, our model predicts that the ordering makes no difference to the final population distribution on an initial population with genotype *i* if, and only if,

$$\mu_{i}P_{x}^{*}P_{y}^{*}=\mu_{i}P_{y}^{*}P_{x}^{*},$$ (1)

where *μ*_*i*_ is the population vector whose *i* ^th^ component is one and all of whose other components are zero.

Supposing we do not know the initial population genotype, we can only guarantee that the order of application is irrelevant when the outcome is the same regardless of the starting genotype. We thus require that $\mu_{i}P_{x}^{*}P_{y}^{*}=\mu_{i}P_{y}^{*}P_{x}^{*}$ for each genotype *i*. Since these unit vectors form a basis of ℝ^*N*^ this occurs precisely when $P_{x}^{*}P_{y}^{*}=P_{y}^{*}P_{x}^{*}$. It follows that drug application will commute precisely when the corresponding limit matrices commute. In practice we may be able to narrow down which genotypes are likely to constitute the population through bacterial genotyping or by observing that certain strains are not viable in the wild due to the high fitness cost of certain mutations.

Mira et al [33] empirically determined the fitness landscapes for *E. coli* in the presence of *N* = 4 resistance–conferring mutations under 15 commonly used antibiotics using average growth rates as a proxy for fitness. We tested commutativity between each pair of these 15 antibiotics and found no commuting pairs. We then tested 10^6^ pairs of random fitness landscapes with varying ruggedness generated according to Kauffman’s NK model for generating “tunably rugged” fitness landscapes [36, 37] using a random neighborhood Boolean function for determining the fitness contributions of each locus. We fixed *N* = 5 and generated each landscape by first drawing *K* uniformly from {0,…,*N* − 1} and then using Kauffman’s model to build a landscape. We found that 0.132% of the landscape pairs generated had limit matrices which commuted, suggesting that commutativity is rare and the order in which drugs are prescribed will be significant in almost all instances.

We now turn our attention to finding antibiotic cycling strategies as considered by Goulart et al [21]. Unless *x* is a flat landscape (taking equal values for all genotypes) there must exist at least one genotype *j* whose fitness is a minimum and which has a fitter neighbor. Such a genotype satisfies ℙ[(*i* → *j*)] = 0 for all genotypes *i*. Hence if *x* is not flat, the limit matrix $P_{x}^{*}$ has at least one column of all zeros and is singular, so there cannot exist a second landscape *y* for which $P_{x}^{*}P_{y}^{*}=I$. Hence for any second landscape *y* there must exist a unit row vector *μ*_*i*_ for which $\mu_{i}P_{x}^{*}P_{y}^{*}\neq \mu_{i}$. This means that natural selection in our model is irreversible, in the sense that for a given (non-flat) landscape we cannot find a second which is guaranteed to reverse its effects unless we first measure the population genotype—a measurement which is non–trivial and not currently common clinical practice. This result precludes the existence of a general cycling strategy that returns the disease population to its original genotype regardless of that starting genotype. If we do in fact know the starting genotype, as we might if the disease is contracted in the wild where resistance–conferring mutations often carry a cost [38] which makes the wild–type genotype most likely, then cycling strategies can be found efficiently by our model. If the initial genotype is known to be *i*, the initial population distribution will be *μ*_*i*_ and a sequence of drugs *X*_1_,…*X*_*k*_ with fitness landscapes *x*_1_,…*x*_*k*_ will constitute a cycling strategy precisely when $\mu_{i}P_{x_{1}}^{*}\ldots P_{x_{k}}^{*}=\mu_{i}$. This criterion will be satisfied when *μ*_*i*_ is a left 1–eigenvector of $P_{x_{1}}^{*}\ldots P_{x_{k}}^{*}$. As such, we can find cycling strategies directly using algebra and avoid the graph–search technique used by Goulart et al [21].

### Evolutionary Steering Can Both Prevent and Promote Resistance

Prescriptions of sequences of drugs occur frequently in the clinic, and often without any guidelines as to which orderings are preferable. Common examples of this include treatment of *H. pylori* [39], Hepatitis B [40] and the ubiquitous change from broad to narrow spectrum antibiotics [41]. The ordering of the sequence is therefore often determined arbitrarily, by the individual clinician’s personal, or historical experience or from laboratory data. However, our model predicts the order in which the drugs are given is likely to have an effect on how the disease evolves and further, once a drug has been given it is not guaranteed that we will be able to reverse the effects. Ideally, we would like to be able to identify drug orderings that lower the probability of a highly resistant disease population emerging during treatment. To consider optimal drug orderings in the context of our model we first need to know the fitness landscapes (or proxies of the fitness landscapes) of a number of antibiotics used to treat a given bacterial infection. Experimentally determining these landscapes requires one to consider all possible 2^*N*^ combinations of genotypes in a set of *N* loci, a task which is prohibitively difficult for all but small values of *N*. De Visser and Krug found that there have been less than 20 systematic empirical studies of fitness landscapes [26] and that landscapes have been calculated for a number of model organisms including *E. coli* [21, 29, 42, 43], *Saccharomyces cerevisiae* [44], *Plasmodium falciparum* [45] and type 1 Human Immunodeficiency Virus [46]. Recent work by Hinkley et al [47], which utilizes regression methods to approximate large fitness landscapes from samples of the space, could help ameliorate the complexity of experimentally determining fitness landscapes.

**Fig 1:**
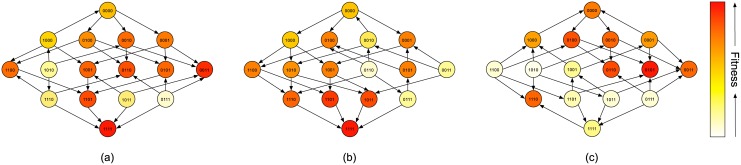
Example fitness landscapes. The evolutionary graphs for the fitness landscapes of *E. coli* with the antibiotics (a) Ampicillin, (b) Ampicillin + Sulbactam and (c) Cefprozil for 4 possible substitutions found in *bla*_TEM-50_. Arrows represent fitness–conferring mutations which can fix under the Strong Selection Weak Mutation assumptions. The absence of an arrow in either direction corresponds to a neutral mutation which cannot fix under these assumptions. Each genotype is shaded according to its fitness normalized to a 0–1 scale.

Mira et al [33] investigated the fitness landscapes of *E. coli* under 15 different *β*-lactam antibiotics using the average growth rates of isogenic populations of each genotype under the drug as a proxy for fitness for a total of *N* = 4 resistance conferring mutations. Fig 1 shows the evolutionary graphs of the fitness landscapes for three of these antibiotics, Ampicillin (Amp), Ampicillin+Sulbactam (Sam) and Cefprozil (Cpr). We will first use these three fitness landscapes to demonstrate the steering hypothesis explicitly. In the case of a single peaked landscape, such as that for Sam, we cannot reduce the likelihood of resistance as all evolutionary trajectories lead to the global fitness optimum. It is only when a drug has a multi–peaked landscape that we may be able to avoid resistance through careful choice of preceding drug. Of the 15 landscapes determined empirically by Mira et al [33] only the landscape for Sam is single peaked. In their review of empirical fitness landscapes, de Visser et al [26] find that biological landscapes show a variable but substantial level of ruggedness, suggesting that multi–peaked landscapes could be quite common.

In the following we take the parameter *r* in Eq (3), which determines how the probability of a mutation fixing is biased by the fitness increase it confers, to be zero. Note that changing the value of *r* will not change the accessibility of an evolutionary trajectory, hence by taking a different value of *r* ≥ 0 we will only change the result quantitatively (the probabilities may change) but not qualitatively. We begin by supposing that we do not know the initial population genotype. This assumption gives us worst case scenario results, and allows pre–existence of any resistant genotype. We model this situation by taking as our prior population distribution *μ* = [1/2^*N*^,…,1/2^*N*^] specifying that each genotype is equally likely to constitute the starting population.

**Fig 2:**
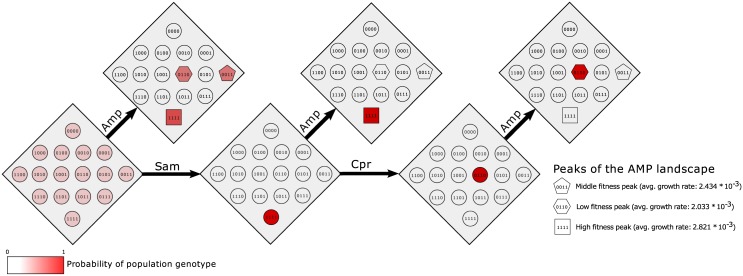
Steering evolution to prevent resistance. The probability distributions for accessibility of the peaks of the Amp landscape for different steering regimes. The initial distribution is *μ* = [1/2^*N*^,…,1/2^*N*^]. When Amp is given first any of the three peaks of the landscape are accessible, with the most resistant genotype 1111 being most likely. If Sam is given first to steer the population to its sole peak 1111, then resistance to Amp will be guaranteed when it is applied. Alternatively, if Sam is given followed by Cpr, then the population evolves to the local optimum genotype 0110 of the Cpr landscape. If Amp is applied to this primed population the global optimum, 1111, is inaccessible.

If we apply the drug Amp to this population distribution we find that in the expected distribution $\mu^{*}=\mu P_{Amp}^{*}$ (shown in the first diamond in the top row of Fig 2) each of the fitness peaks can be found. In particular, the most highly resistant genotype 1111 can arise in the population with probability 0.62. Suppose instead we apply Sam first. In this case as the landscape is single peaked the population will converge to the global optimum genotype 1111. This genotype is also the global optimum of the Amp landscape and hence if we apply Amp after Sam we will encounter high resistance. We have steered the population with one drug to a configuration which increases the probability of resistance to a second. Next suppose that we instead apply Cpr after Sam; in this case the population is guaranteed to evolve to a local optimum 0110 of the Cpr landscape. 0110 is the least fit local optimum of the Amp landscape. Thus if we apply Amp to the population primed by Sam → Cpr then evolution to the global optimum 1111 is not possible. This example demonstrates the steering hypothesis, that evolution can be shepherded through careful orderings of multiple drugs to increase or decrease the likelihood of resistance emerging.

To test our steering hypothesis more rigorously, we performed an *in silico* test of steering using combinations of one, two or three preceding drugs for each of the 15 drugs for which we know the landscapes. Table 1 shows, for each of the 15 antibiotics, the steering combinations predicted to minimize the probability of evolution to the most resistant genotype in the landscape of that antibiotic when applied in order before it. Again we took *μ* = [1/2^*N*^,…,1/2^*N*^] to model the worst case scenario for pre–existing resistance. We found that for 3 of the 15 drugs there exists another which steers the initial population *μ* to a configuration from which evolution to the global fitness optimum of the drug landscape is prevented entirely. This number rose to 6 when pairs of drugs applied sequentially are used to steer the population and to 7 when triples were applied in sequence.

**Table 1:** Steering sequences which minimize the probability of evolution to the highest peak of the landscape.

**Final drug**	**# of Peaks**	**Fittest peak genotype**	**Probability of ending at highest fitness genotype (no steering)**	**Best single steering drug**	**Probability of ending at highest fitness genotype (with best single steering drug)**	**Best ordered pair of steering drugs**	**Probability of ending at highest fitness genotype (with best steering pair)**	**Best ordered triple of steering drugs**	**Probability of ending at highest fitness genotype (with best steering triple)**
Ampicillin (AMP)	3	1111	0.62	CPR	0.25	AMP → CPR	0.0	-	-
Amoxicillin (AMX)	2	1101	0.75	CTX	0.62	SAM → CPR	0.51	CRO → SAM → CPR	0.51
Cefaclor (CEC)	3	0011	0.19	SAM	0.0	-	-	-	-
Cefotaxime (CTX)	4	1111	0.17	CPR	0.04	AMP → CPR	0.0	-	-
Ceftizoxime (ZOX)	2	0111	0.86	AMX	0.81	SAM → AMX	0.75	CEC → CPR → AMC	0.75
Cefuroxime (CXM)	2	0111	0.56	AMC	0.33	CXM → AMC	0.19	AMC → CXM → AMC	0.11
Ceftriaxone (CRO)	4	1111	0.28	CEC	0.20	TZP → CEC	0.05	AMC → TZP → CEC	0.0
Amoxicillin +Clav (AMC)	2	1101	0.67	CXM	0.39	AMC → CXM	0.23	CXM → AMC → CXM	0.13
Ceftazidime (CAZ)	3	0110	0.39	TZP	0.08	AMC → TZP	0.0	-	-
Cefotetan (CTT)	5	0111	0.18	AMC	0.0	-	-	-	-
Ampicillin +Sulbactam (SAM)	1	1111	1.0	*	1.0	*	1.0	*	1.0
Cefprozil (CPR)	3	0101	0.25	AMP	0.0	-	-	-	-
Cefpodoxime (CPD)	2	1111	0.81	CRO	0.74	CTX → FEP	0.57	SAM → CEC → CRO	0.50
Piperacillin +Tazobactam (TZP)	2	0101	0.80	CTT	0.72	CTT → CTT	0.72	CTX → FEP → CTT	0.68
Cefepime (FEP)	4	1111	0.48	CTX	0.39	CAZ → CEC	0.36	CAZ → CEC → CTX	0.33

For each of the 15 antibiotic landscapes we have derived the (ordered) sets of one, two and three steering drugs which minimize the probability of evolution to the global fitness peak of that landscape. In the case that an ordered set of steering drugs reduced the probability to 0 we have not considered ordered sets of greater length (marked as—in the table).

* As the landscape for SAM is single–peaked there can be no combination of steering drugs which reduce the probability of finding the global optimum. In all experiments the initial population distribution is taken as *μ* = [1/2^*N*^,…,1/2^*N*^] and *r* = 0.

We then performed a second *in silico* experiment to find combinations of steering antibiotics maximizing the probability that evolution proceeds to the least fit local optima in the landscape of a final antibiotic (Table 2). We found that, excluding the single peaked landscape for Ampicillin with Sulbactam, there exist 0 drugs for which a single other drug is able to steer the population to a configuration from which evolution to only the least fit optimum is possible. If pairs of drugs are used to steer there are 3 such drugs (including the example presented in the above demonstration) and if triples of steering drugs are considered there remains only 3. These findings suggest that through careful choice of steering drugs we may be able to prevent the emergence of resistance. During these experiments we found that 14 of the 15 antibiotics in our experiment (Cefpodoxime (CPD) being excluded) appeared in an optimal steering combination of some length.

**Table 2:** Steering sequences which maximize the probability of evolution to the lowest peak of the landscape.

**Final drug**	**# of Peaks**	**Least fit peak genotype**	**Probability of ending at lowest fitness genotype (no steering)**	**Best single steering drug**	**Probability of ending at lowest fitness genotype (with best single steering drug)**	**Best ordered pair of steering drugs**	**Probability of ending at lowest fitness genotype (with best steering pair)**	**Best ordered triple of steering drugs**	**Probability of ending at lowest fitness genotype (with best steering triple)**
Ampicillin (AMP)	3	0110	0.19	CPR	0.51	SAM → CPR	1.0	-	-
Amoxicillin (AMX)	2	0010	0.25	CTX	0.38	SAM → CPR	0.49	AMP → SAM → CPR	0.49
Cefaclor (CEC)	3	0100	0.41	TZP	0.70	CXM → AMC	0.78	AMC → CXM → AMC	0.87
Cefotaxime (CTX)	4	1010	0.21	FEP	0.27	CTX → FEP	0.43	SAM → CEC → CRO	0.50
Ceftizoxime (ZOX)	2	1001	0.14	AMX	0.19	SAM → AMX	0.25	AMP → SAM → AMX	0.25
Cefuroxime (CXM)	2	0100	0.44	AMC	0.67	CXM → AMC	0.81	AMC → CXM → AMC	0.89
Ceftriaxone (CRO)	4	0100	0.30	CXM	0.59	AMC → CXM	0.76	CXM → AMC → CXM	0.86
Amoxicillin +Clav (AMC)	2	0100	0.33	CXM	0.61	AMC → CXM	0.77	CXM → AMC → CXM	0.87
Ceftazidime (CAZ)	3	0011	0.26	FEP	0.28	CAZ → CEC	0.40	CAZ → AMX → FEP	0.45
Cefotetan (CTT)	5	1101	0.19	AMX	0.75	SAM → AMX	1.0	-	-
Ampicillin +Sulbactam (SAM)	1	1111	1.0	*	1.0	*	1.0	*	1.0
Cefprozil (CPR)	3	0011	0.25	ZOX	0.26	CAZ → CEC	0.34	CAZ → CEC → FEP	0.43
Cefpodoxime (CPD)	2	1010	0.19	CRO	0.26	CTX → FEP	0.43	SAM → CEC → CRO	0.50
Piperacillin +Tazobactam (TZP)	2	1000	0.20	CTT	0.28	CTT → CTT	0.28	CTX → FEP → CTT	0.32
Cefepime (FEP)	4	0000	0.14	FEP	0.14	TZP → CEC	0.18	CXM → AMC → CXM	0.20

For each of the 15 antibiotics landscapes we have derived the (ordered) sets of one, two and three steering drugs which maximize the probability of evolution to the least fit peak of that landscape. In the case that an ordered set of steering drugs increases the probability to 1 we have not considered ordered sets of greater length (marked as—in the table).

* As the landscape for SAM is single peaked there can be no combination of steering drugs which change the probability of finding the global optimum. In all experiments the initial population distribution is taken as *μ* = [1/2^*N*^,…,1/2^*N*^] and *r* = 0.

Whilst careful selection of drugs for steering can prevent the emergence of resistance, arbitrary drug orderings can also promote it. We performed an exhaustive *in silico* search of all singles, pairs, and triples of steering drugs applied sequentially to prime the initial population *μ* for a final application of each of the 15 antibiotics (Table 3). We found that steering with a single drug increased the likelihood of the most resistant genotype emerging in 57.3% of cases and decreased the likelihood in 29.8% of cases. Steering with pairs of drugs increased the likelihood in 64.1% of cases and decreased it in 28.4% of cases and steering with triples increased the likelihood in 65.6% of cases and decreased it in 27.5%. We tested the robustness of these results to changes in the value of *r* in Eq (3) and found that each of these values are changed by less than 2% for *r* = 1. For *r* → ∞ we found that 56.0%, 68.1% and 71.2% of singles, doubles and triples (respectively) of steering drugs increased the likelihood of the most highly resistant genotype being found whereas only 22.2%, 20.0% and 19.2% of singles, pairs and triples (respectively) decreased it.

**Table 3:** Analysis of all possible steering sequences of length one, two and three.

**Final drug**	# of single steering drugs better (*/15)	# of single steering drugs worse (*/15)	# of steering pairs better(*/225)	# of steering pairs worse (*/225)	# of steering triples better (*/3375)	# of steering triples worse (*/3375)
Ampicillin (AMP)	7 (46.7%)	7 (46.7%)	97 (43.1%)	126 (56.0%)	1373 (40.7%)	1998 (59.2%)
Amoxicillin (AMX)	5 (33.3%)	9 (60.0%)	60 (26.7%)	164 (72.9%)	787 (23.3%)	2578 (76.4%)
Cefaclor (CEC)	9 (60.0%)	5 (33.3%)	132 (58.7%)	88 (39.1%)	2087 (61.8%)	1264 (37.5%)
Cefotaxime (CTX)	4 (26.7%)	10 (66.7%)	67 (29.8%)	155 (68.9%)	989 (29.3%)	2335 (69.2%)
Ceftizoxime (ZOX)	2 (13.3%)	12 (80.0%)	31 (13.8%)	193 (85.8%)	444 (13.2%)	2930 (86.8%)
Cefuroxime (CXM)	5 (33.3%)	9 (60.0%)	95 (42.2%)	128 (56.9%)	1486 (43.5%)	1885 (55.9%)
Ceftriaxone (CRO)	5 (33.3%)	9 (60.0%)	61 (27.1%)	163 (72.4%)	810 (24.0%)	2564 (76.0%)
Amoxicillin +Clav (AMC)	7 (46.7%)	7 (46.7%)	99 (44.0%)	124 (55.1%)	1428 (42.3%)	1943 (57.6%)
Ceftazidime (CAZ)	4 (26.7%)	10 (66.7%)	76 (33.8%)	147 (65.3%)	1218 (36.1%)	2153 (63.8%)
Cefotetan (CTT)	4 (26.7%)	10 (66.7%)	58 (25.8%)	166 (73.8%)	843 (25.0%)	2531 (75.0%)
Ampicillin +Sulbactam (SAM)	0 (0.0%)	0 (0.0%)	0 (0.0%)	0 (0.0%)	0 (0.0%)	0 (0.0%)
Cefprozil (CPR)	7 (46.7%)	7 (46.7%)	113 (50.2%)	110 (48.9%)	1703 (50.5%)	1668 (49.4%)
Cefpodoxime (CPD)	3 (20.0%)	11 (73.3%)	26 (11.6%)	195 (86.7%)	282 (8.4%)	3077(91.2%)
Piperacillin +Tazobactam (TZP)	2 (13.3%)	12 (80.0%)	11 (5.9%)	213 (94.7%)	81 (2.4%)	3293 (97.6%)
Cefepime (FEP)	3 (20.0%)	11 (73.3%)	34 (15.1%)	190 (84.4%)	402 (11.9%)	2972 (88.1%)
Overall	67 (29.8%)	129 (57.3%)	960 (28.4%)	2162 (64.1%)	13933 (27.5%)	33200 (65.6%)
Overall (Without steering for SAM)	67 (31.9%)	129 (61.4%)	960 (30.5%)	2162 (68.6%)	13933 (29.5%)	33200 (70.3%)

For each of the 15 antibiotics we calculated the probability evolution to the most resistant genotype to that drug when that drug is applied to a population first primed by a single, ordered pair or ordered triple of drugs. This table shows the number of priming singles, pairs and triples which improve or worsen the likelihood of evolution to that most highly resistant genotype. We allowed steering drugs to appear multiple times in the combination and also allowed the final drug to appear as a steering drug. In each case the initial population was given by *μ* = [1/2^*N*^,…,1/2^*N*^] and *r* = 0. As the SAM landscape is single-peaked no combination of steering drugs will improve or worsen the outcome. As such, we have computed the overall numbers both with and without the contribution of the SAM row.

For each of the antibiotics, except Cefaclor, Cefprozil and Ampicillin+Sulbactam (which is single peaked making steering irrelevant), we found that a random steering combination of length one, two or three is more likely to increase the chances of resistance than to reduce it. Indeed, for Piperacillin+Tazobactam and Ceftizoxime we found that a random steering combination will increase the probability of the most highly resistance genotype occurring in more than 80% of cases, suggesting that sequential multidrug treatments which use these very common antibiotics should proceed with caution. These findings suggest that the present system of determining sequential drug orderings without quantitative optimization based guidelines could in fact be promoting drug resistance and that to avoid resistance we must carefully consider the order in which drugs are applied.

## Discussion

We have reduced the evolutionary dynamics of an asexually reproducing population to a biased random walk on a fitness landscape. Through this reduction we explored the evolutionary trajectories of a population by considering the algebraic properties of the Markov chain transition matrix associated with the random walk. We have demonstrated that evolution on fitness landscapes is non-commutative, in the sense that the same drugs applied in different orders can result in different final population configurations, through parallels with the non-commutativity of matrix multiplication. Further, we demonstrated that it is possible to find sequences of drugs that can be applied to both avoid and promote the emergence of resistance. In particular, we performed an exhaustive analysis of the evolutionary trajectories of *E. coli* under short drug sequences (two to four drugs) chosen from 15 *β*–lactam antibiotics using empirically determined fitness landscapes and found that the majority, approximately 70%, of sequential treatments with 2–4 drugs increase the likelihood of resistance emerging.

In light of the slow pace of novel antibiotic discovery and the rapid emergence of resistance to the presently most utilized antibiotics, these findings suggest a new treatment strategy—one in which we use a sequence of drugs (or even treatment breaks which themselves impose a selective pressure [38]) to steer, in an evolutionary sense, the disease population to avoid resistance from developing. Further, the drugs used to prime the disease population for treatment by an effective antibiotic do not themselves need to be the most effective drugs available. This means that there could be a large pool of potential steering drugs in the form of antibiotics which have gone unused for many years due to their inefficacy as a single agent. However, in the same way that the drug ordering can be used to steer away from resistance we have shown it can also be used to make resistance more likely. Our results show that we may be inadvertently selecting for highly resistant disease populations through arbitrary drug ordering in the same way that highly resistant disease can emerge through irresponsible drug dosing. The most striking example is that of Piperacillin with Tazobactam, a drug often prescribed in a hospital setting after others fail, which has an increased likelihood of resistance when prescribed after a pair or triple of others drugs in over 90% of cases. If we are to avoid resistance to our most effective drugs we must carefully consider how they are used together with other drugs, both in combination and in sequence, and take appropriate steps to reduce the risk.

A major difficulty in using sequential drug treatments to steer disease populations is that in order to predict the outcomes we must know the fitness landscapes of the drugs involved. De Visser and Krug [26] state that there exist fewer than 20 systematic studies of fitness landscapes and that these studies consider between 3 and 9 possible mutations. For steering to be fully effective we must account for all likely fitness conferring mutations and their effects on fitness under many drugs. Thus, many of the studies reviewed by de Visser and Krug are insufficient for determining clinically actionable steering strategies for certain diseases. Fortunately, for a number of highly resistant infectious diseases [48, 49] and cancers [50, 51], only a small number of mutations appear to contribute to resistance. Further, recent work by Hinkley et al [47] in HIV has introduced a method to approximate large fitness landscapes from relatively fewer data points using a regression method. It follows that determining the landscapes is not an entirely intractable problem. A further complication in determining steering strategies is that fitness landscapes can be dependent on the disease microenvironment and have the potential to change from patient to patient or throughout the course of treatment. The consequences of such effects on fitness landscapes have not yet been experimentally determined.

Two major assumptions within our modeling are that drugs are administered for sufficiently long that evolution can converge to a local fitness optimum and that this convergence is guaranteed to occur. This assumption poses two potential problems in converting our model predictions to predictions of real–world bacterial evolution. The first is that if selection is strong and mutations are rare then there is a possibility of the population being driven to extinction before an adaptive mutation occurs. We have chosen to ignore this possibility within our modeling as in the context of treating bacterial infections this would constitute a success. The second is that the time to convergence could be prohibitively long for steering to constitute a realistic treatment strategy. We believe that the assumption of reasonable convergence times could be valid as adaptive walks in rugged landscapes are often short [52]. However, it has been shown that for certain landscapes there can exist adaptive walks of length exponential in the number of loci [53], but as we get to choose those drugs with which to steer we can avoid landscapes for which the convergence time is prohibitively long. Further, our model is not necessarily restricted to the dynamics within a single patient. Goulart et al [21] used fitness landscapes to explore whole hospital scale antibiotic treatment strategies and our model, as an encoding of evolution on fitness landscapes, is capable of making predictions at this scale as well. As such, even if evolutionary convergence is experimentally determined to be prohibitively slow for steering to be effective as a treatment for bacterial infection within a single patient, our results will still hold in scenarios which admit longer timescales. Specifically, evolutionary steering could provide an effective means to avoid the emergence of drug resistance at the hospital scale or in long–term diseases such as HIV or Tuberculosis.

The Strong Selection Weak Mutation model we have used here is a highly simplified, yet well studied model of evolution. The model ignores much of the complexity of the evolutionary process, specifically simplifying the genotype–phenotype map, ignoring the disease microenvironment and making the assumption of a monomorphic disease population in which deleterious and neutral mutations cannot fix. Under certain regimes of population size and mutation rates these simplifying assumptions break down [54]. For example, if the population is sufficiently large then stochastic tunneling [55]—where double mutations can occur allowing the crossing of fitness valleys—can arise causing a breakdown of the Strong Selection assumption. Similarly, if the mutation rate is sufficiently high then the population ceases to be monomorphic and forms a quasispecies [56, 57]. Conversely, if the population is sufficiently small then it becomes possible for deleterious mutations to fix [58–60]. Finally, we have ignored the possibility of neutral spaces in the fitness landscape which have been shown to have significant impact on whether non-optimal genotypes can fix in the population as well as the time taken for evolution to find a locally optimal genotype [61, 62]. The only neutral mutation present in the empirical landscapes we use in this work is in the single–peaked landscape for Ampicillin+Sulbactam (Fig 1). As the landscape is single peaked, omitting this mutation does not prevent any evolutionary trajectories. Thus, the results of our exhaustive search of sequential treatments are unaffected by the assumption that neutral mutations cannot fix. We believe that each of the possible breakdowns of the SSWM model will have important consequences for the possibility and efficacy of steering, especially as larger landscapes are considered. However, a proper treatment of their implications is beyond the scope of this paper. In our future work we aim to undertake a comprehensive study of the implications of population size, mutation rate, neutral drift and evolutionary convergence times on our steering hypothesis.

To further develop the theory of evolutionary steering as a clinically viable strategy for preventing or treating highly resistant disease we must begin to collect data regarding empirical landscapes. The data cannot be collected at a large scale by the existing method of engineering all genotypes of interest and testing their fitness. Such experiments are intractable for all but the smallest landscapes. Instead we must begin to measure and collect the genotypes and fitness of pathogens that appear in the clinic. Hinkley et al [47] attempted to reconstitute the empirical landscapes for HIV-1 under different drugs which were later analyzed by Kouyos et al [63]. This analysis was only possible due to the availability of a data set of over 70,000 clinical samples of HIV-1 with recorded values of fitness under a number of antiretroviral drugs. Such data sets for bacterial pathogens are not yet available but should become easier to obtain as the cost of genome sequencing continues to fall. Once the data are available we will be able test the validity of many of the assumptions of our model. Such a data set will also have many uses beyond the work presented here, for example in tracking the spread and evolutionary history of highly resistant disease through phylodynamics [64].

The model presented here is a simplification of the evolutionary process; however, given that non-commutativity is present in this highly simplified model, it is unlikely that commutativity will emerge as more complexity is introduced. It follows that the cautionary message regarding sequential drug application which results from our simplified model merits serious consideration. Whether or not measuring fitness landscapes provides sufficient information to correctly identify, or to serve as a heuristic in identifying, optimal drug orderings *in vivo* is a question that cannot be answered through mathematical modeling alone. It is only by verifying the predictions of steering strategies given by our model through biological experiment that we can determine whether they are viable. Supposing our model predictions are indeed viable, then knowledge of some approximation to the fitness landscapes of the presently most used antibiotics could, in combination with our model, provide at least a good heuristic for how to proceed with multi-drug treatments, future antibiotic stewardship programs and clinical trial design.

## Materials and Methods

### Evolution on Fitness Landscapes

**Fig 3:**
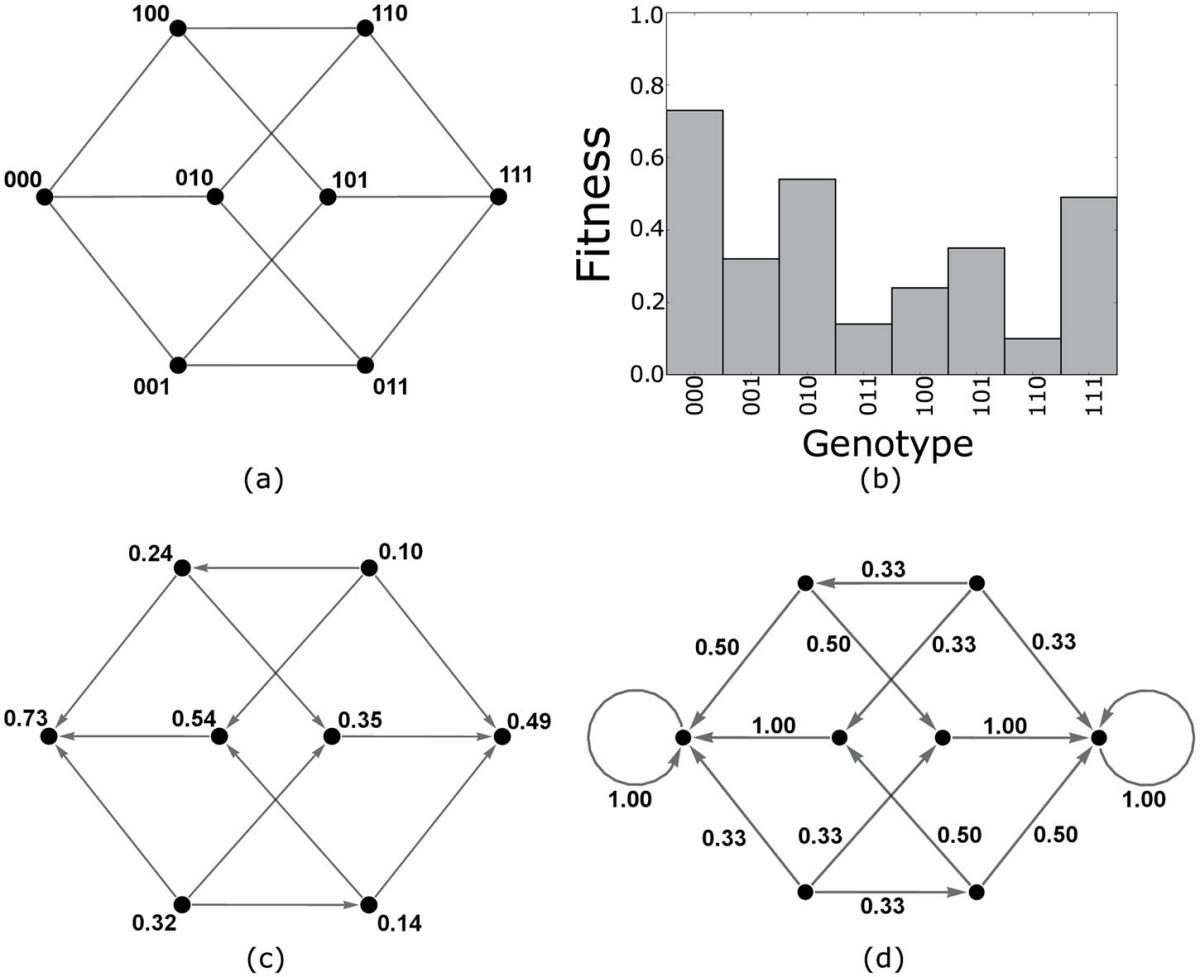
Constructing the Markov chain from a fitness landscape. (a) The space of genotypes comprising bit strings of length *N* = 3. The vertices represent genotypes, and edges connect those genotypes that are mutational neighbors. (b) An example fitness landscape. (c) The directed evolutionary graph according to the landscape in (b) where the vertices represent genotypes and are labeled by the associated fitness. The directed graph edges are determined by the fitness function and represent those mutations which can fix in a population (those which confer a fitness increase). (d) The Markov chain constructed for the same landscape according to Eqs (2) and (3) with *r* = 0.

We begin with the concept of a fitness landscape introduced by Wright [59] and used by Weinreich et al [27] and Tan et al [29] to study evolutionary trajectories in asexually reproducing populations. We represent the genotype of an organism by a bit string of length *N* and model mutation as the process of flipping a single bit within this string. This model of mutation only accounts for point mutations and ignores the possibility of other biologically relevant mutations such as gene insertions, gene deletions and large structural changes to the genotype. This gives a set of 2^*N*^ possible genotypes in which individuals of a given genotype, say *g*, can give rise to mutated offspring which take genotypes given by one of the *N* mutational neighbors of *g*—precisely those genotypes *g*′ for which the Hamming distance [65], Ham(*g*, *g*′), from *g* is 1. As such, our genotype space can be represented by an undirected *N*-dimensional (hyper–)cube graph with vertices in {0,1}^*N*^ representing genotypes and edges connecting mutational neighbors (Fig 3a).

We define a selective pressure on our graph that drives evolution, for example through an environmental change or drug application, as a fitness function

$$f:{\left\{0,1\right\}}^{N}\to R^{\geq 0}.$$ (2)

This fitness function represents a genotype-phenotype map in the simplest sense—assigning to each genotype a single real-valued fitness. Gillespie [34, 35] showed that if the mutation rate *u* and population size *M* of a population satisfy *Mu* log*M* ≪ 1, and if we assume that each mutation is either beneficial or deleterious, then each beneficial mutation in the population will either reach fixation or become extinct before a new mutation occurs. Further, selection will be sufficiently strong that any deleterious mutation will become extinct with high probability and hence we may assume that this always occurs. In the case that *Mu* ^2^ ≈ 1 stochastic tunneling [55, 66, 67] through double mutations can occur and we cannot ignore deleterious mutations. Assuming *Mu* log*M* ≪ 1, then after each mutation the population will stabilize to consist entirely of individuals with the same genotype and this genotype will be eventually replaced by a fitter neighboring genotype whenever one exists. This observation gives rise to the Strong Selection Weak Mutation (SSWM) model, which models a population as isogenic and occupying a single vertex on a directed graph on the set of 2^*N*^ possible genotypes, {0,1}^*N*^, in which there exists an edge from vertex *a* to a neighboring vertex *b* if, and only if, *f*(*b*) > *f*(*a*) (see Fig 3b and 3c). This population undergoes a stochastic walk on the graph in which the population genotype is replaced by a fitter adjacent genotype with some probability. In fact, this model still holds in the case that *Mu* ≫ 1 ≫ *Mu* ^2^ [26]. Several ‘move rules’ have been proposed which can be used to select an adjacent fitter neighbor during this stochastic walk [52] and which of these move rules is most accurate depends on the population size [26]. Common move rules include selecting the fittest neighbor [36, 68], selecting amongst fitter neighbors at random [69–71] or selecting fitter neighbors with probability proportional to the fitness increase conferred [34, 35, 72]. We encapsulate each of these variants of the SSWM model within our model.

### A Markov Model of Evolution

The SSWM model of evolution reduces the evolutionary process to a random walk on a directed graph and hence can be modeled by a Markov chain [73]. For a fitness function *f*:{0,1}^*N*^ → ℝ ^≥ 0^ we can define a transition matrix *P* = [ℙ(*i* → *j*)]_*i*,*j* ∈ {0,1}^*N*^_ for a time–homogeneous absorbing Markov Chain by setting, for *i* ≠ *j*,

$$\mathbb{P}\left(i\to j\right)=\{\begin{array}{ll} \frac{{\left(f\left(j\right)-f\left(i\right)\right)}^{r}}{\sum_{\begin{array}{c} g\in {\left\{0,1\right\}}^{N},\text{ Ham}\left(i,g\right)=1\\ f\left(g\right)-f\left(i\right)>0 \end{array}}{\left(f\left(g\right)-f\left(i\right)\right)}^{r}} & \text{ if }f\left(j\right)>f\left(i\right)\text{ and Ham}\left(i,j\right)=1\\ 0 & \text{otherwise} \end{array}$$ (3)

and

$$P\left(i\to i\right)=\{\begin{array}{cc} 1 & \text{if}\;i\;\text{has}\;\text{no}\;\text{fitter}\;\text{one-step}\;\text{mutational}\;\text{neighbors}\\ 0 & \text{otherwise} \end{array}$$ (4)

for each *i* (see Fig 3d). Here the parameter *r* ≥ 0 determines the extent to which the fitness increase of a mutation affects its likelihood of determining the next population genotype. In the case *r* = 0, we have the random move SSWM model (as in [69, 69, 71]), in the limit *r* → ∞ we have the steepest gradient ascent SSWM model (as in [36, 68]), and for *r* = 1 we have probability proportional to fitness increase (as in [34–36]). In the case *f*(*i*) = *f*(*j*) the mutation is neutral and, under the assumptions of SSWM, cannot fix. Thus, this model differs from the Markov model used by [74] to study the neutral theory of evolution as we do not allow neutral or deleterious mutations to fix in the population.

Using this Markov chain we can explore the possible evolutionary trajectories of a population on a given fitness landscape *f*. We next define a collection of population row vectors *μ* ^(*t*)^ for each *t* ∈ ℕ, where *μ* ^(*t*)^ has length 2^*N*^ and *k* ^th^ component which gives the probability that the population has the *k* ^th^ genotype at time *t* (where the genotypes are ordered numerically according to their binary value). These time steps *t* are an abstraction which discretely measure events of beneficial mutations occurring and fixing in the population. As such, the actual time between steps *t* and *t*+1 is not constant but may be considered drawn from a distribution parameterized by the mutation rate, reproductive rate and the number of beneficial mutations that can occur. This distribution could, for example, be determined by a Moran [58] or Wright–Fisher [59, 60] process depending on how we choose to interpret the fitness values given by *f*. If the population has a genotype corresponding to a local optimum of the fitness landscape at time *t* then there are no beneficial mutations that can occur and this definition of a time step is not well defined. In this case there can be no more changes to the population under the SSWM assumptions and for mathematical convenience we define the probability of a local optimum population genotype remaining unchanged as 1 in Eq 3 to ensure our model is a Markov Chain. In this case the step *t* to *t*+1 can be chosen to take some fixed arbitrary time.

The distribution of a population at time *t* is related to its initial distribution, *μ* ^(0)^, by

$$\mu^{\left(t\right)}=\mu^{\left(0\right)}P^{t}.$$ (5)

Since the Markov chain is absorbing we know that there exists some *k* such that *P* ^*k*^ *P* = *P* ^*k*^ [73]. Consequently, we know that the matrix

$$P^{*}=\lim_{t\to \infty }P^{t}$$ (6)

exists and in fact this limit is reached after only finitely many matrix multiplications. To intuitively see that this limit is reached in finitely many steps note that all paths through the Markov chain are strictly increasing in fitness and there are only finitely many states (corresponding to the genotypes). Thus a given initial population distribution *μ* ^(0)^ will converge to a stationary distribution μ* after a finite number of steps in our model. Furthermore, if P* is known then we compute the stationary distribution μ* as

$$\mu^{*}=\mu^{\left(0\right)}P^{*}.$$ (7)

In particular, provided a drug is applied for sufficiently long to ensure that the disease population reaches evolutionary equilibrium, we can explore the effects of applying multiple drugs sequentially by considering the matrices P* for the associated fitness landscapes. By encoding the evolutionary dynamics in a Markov chain we can investigate the evolutionary process from an algebraic perspective. In particular, as the transition matrix *P* encodes all of the evolutionary dynamics of the associated fitness landscape *f*, we can explore global properties of *f* by considering the algebraic properties of *P*.
